# Death of Dr. Henry Campbell

**Published:** 1892-01

**Authors:** 


### DEATH OF DR. HENRY CAMPBELL.

"AUGUSTA, GA., December 15.

"Dr. Henry Frazer Campbell died at 10:30 o'clock tonight after a long illness which commenced last February. He was unconscious the last thirty-six hours and passed off quietly. Dr. Campbell was a leading physician of Augusta, a most eminent and skilled doctor with a world-wide reputation. He was known in Europe as well as in America. Dr. Campbell was born in Savannah, Ga., February 10, 1824, sixty-seven years ago.

"He was the discoverer of the over-excito motory system, although Drs. Marshall, Hall, and Claude Benton, of London, at first disputed Dr. Campbell's claim of this discovery, but finally admitted Dr. Campbell made the discovery two years previous to them. Dr. Campbell was honorary fellow to the Imperial Academy of Medicine at St. Petersburg, Russia, a member of the American and Southern Gynecological Society, and in 1885 was honored and held the office of president of the American Medical Association. Dr. Campbell was one of Augusta's most prominent and respected citizens and his death will be deeply mourned. He leaves a wife and an only child, Mrs. Carrie Doughty. He was known by every doctor in this country and was connected with every medical association."

It is with sadness and sorrow that we transfer the above notice to our columns. The above sketch is but an imperfect portraiture of the career of a brilliant genius in medicine and surgery—a man who drank deep at all the fountains of our professional learning.

Science had no more devoted son, and bestowed upon him all her choicest laurels. He won honors in all the highest courts of earth where our noblè and exalted profession had attained to highest renown.

Yes!—his honors were world wide; and his fame must live brightly forever in the hearts and minds of all those who worship at the shrine of our exalted profession. But a brighter and lovlier page ever illuminated the charming book of his long and illustrious life.

It glowed with the sunshine of a noble and tender disposition, a warm and genial heart, which reached out and included all his fellow men and especially all those who came within the range of his grand life work. His disposition was genial and bright, his friendship warm and tender, and his heart was gold with sweet tendernesss like that of a woman's. While the whole profession will mourn him, none will do so with more heartfelt grief than those who felt the tender touch of his bracing hand, while young and inexperienced in the profession, as did the writer. There was a charm and grace about his deportment towards students and younger members of his profession, that those who witnessed it can never forget.

Yes!—He was an honor to his profession; he was an honor to humanity and to his native State. He was an humble trusting Christian, and a royal gentleman at all times and under all circumstances.

I love to recall his kindness to me, but I cannot speak of it wtihout being overcome by my feelings of grateful recognition; and here I roll down the curtain, dropping a tear to his memory, but shall love to recall all his kindness to me, and his great nobility of soul through all the years that God may allot me on this earth.

We extend our heartfelt sympathies to his noble wife and daughter in their sad affliction. D. H. H.

